# Pulmonary Microvascular Albumin Leak Is Associated with Endothelial Cell Death in Murine Sepsis-Induced Lung Injury *In Vivo*

**DOI:** 10.1371/journal.pone.0088501

**Published:** 2014-02-07

**Authors:** Sean E. Gill, Ravi Taneja, Marta Rohan, Lefeng Wang, Sanjay Mehta

**Affiliations:** 1 Pulmonary Inflammation, Injury, and Repair Lab (PIIRL), Centre for Critical Illness Research, Lawson Health Research Institute, London Health Sciences Center, London, Ontario, Canada; 2 Division of Respirology, Department of Medicine, Schulich School of Medicine and Dentistry, Western University, London, Ontario, Canada; 3 Department of Physiology and Pharmacology, Schulich School of Medicine and Dentistry, Western University, London, Ontario, Canada; 4 Department of Critical Care Medicine, Schulich School of Medicine and Dentistry, Western University, London, Ontario, Canada; 5 Department of Anesthesia and Perioperative Medicine, Schulich School of Medicine and Dentistry, Western University, London, Ontario, Canada; University of Illinois College of Medicine, United States of America

## Abstract

Sepsis is a systemic inflammatory response that targets multiple components of the cardiovascular system including the microvasculature. Microvascular endothelial cells (MVEC) are central to normal microvascular function, including maintenance of the microvascular permeability barrier. Microvascular/MVEC dysfunction during sepsis is associated with barrier dysfunction, resulting in the leak of protein-rich edema fluid into organs, especially the lung. The specific role of MVEC apoptosis in septic microvascular/MVEC dysfunction *in vivo* remains to be determined. To examine pulmonary MVEC death *in vivo* under septic conditions, we used a murine cecal ligation/perforation (CLP) model of sepsis and identified non-viable pulmonary cells with propidium iodide (PI) by intravital videomicroscopy (IVVM), and confirmed this by histology. Septic pulmonary microvascular Evans blue (EB)-labeled albumin leak was associated with an increased number of PI-positive cells, which were confirmed to be predominantly MVEC based on specific labeling with three markers, anti-CD31 (PECAM), anti-CD34, and lectin binding. Furthermore, this septic death of pulmonary MVEC was markedly attenuated by cyclophosphamide-mediated depletion of neutrophils (PMN) or use of an anti-CD18 antibody developed for immunohistochemistry but shown to block CD18-dependent signaling. Additionally, septic pulmonary MVEC death was iNOS-dependent as mice lacking iNOS had markedly fewer PI-positive MVEC. Septic PI-positive pulmonary cell death was confirmed to be due to apoptosis by three independent markers: caspase activation by FLIVO, translocation of phosphatidylserine to the cell surface by Annexin V binding, and DNA fragmentation by TUNEL. Collectively, these findings indicate that septic pulmonary MVEC death, putatively apoptosis, is a result of leukocyte activation and iNOS-dependent signaling, and in turn, may contribute to pulmonary microvascular barrier dysfunction and albumin hyper-permeability during sepsis.

## Introduction

Sepsis remains a common and important clinical problem with significant morbidity and mortality. Sepsis is the most common cause of mortality in the contemporary Intensive Care Unit (ICU) and has a mortality of 30–40% [1], [2]. In North America, ∼one million cases of sepsis occur annually, leading to severe sepsis 40% of the time and 300,000 deaths. This consumes up to 45% of total ICU costs [2], [3]. Morbidity/mortality in sepsis are largely due to multiple organ dysfunction/failure, most commonly lung injury, as well as renal and cardiac dysfunction [2]–[6]. Despite intensive basic and clinical research, treatment of sepsis and related organ dysfunction consists largely of supportive care, as all novel anti-inflammatory therapeutic approaches, including the recently withdrawn activated protein C, have failed to improve the outcome of patients with sepsis and multiple organ dysfunction [6]–[8].

Septic organ dysfunction is due, in part, to an overwhelming systemic inflammatory process, characterized by the activation of both circulating (e.g. Polymorphonuclear [PMN] leukocytes) and tissue-resident inflammatory cells (e.g. macrophages), as well as the enhanced production and release of a plethora of soluble inflammatory mediators, including lipopolysaccharide (LPS) and various cytokines (e.g. tumour necrosis factor [TNF] α, interleukin [IL] 1β). It is increasingly recognized that septic organ dysfunction is also due to significant perturbations in vascular function, including both disturbed systemic hemodynamics with global changes in blood flow, and more importantly, abnormal function of the microvasculature of many organs. Microvascular dysfunction is characterized by impaired barrier function with increased permeability leading to extra-vascular leak of protein-rich edema and PMN influx into organs [9]–[14], microvascular thrombosis [15], [16], and impaired distribution of blood flow in microvascular beds [17], [18]. Microvascular dysfunction is clinically important, as it has been documented early in the course of sepsis in humans, and is associated with increased mortality [19], [20], especially if it persists over time [21].

Microvascular endothelial cells (MVEC) are critical modulators of blood flow and microvascular function in individual organs. Furthermore, microvasculature and MVEC are principal targets of the overwhelming systemic inflammation of sepsis [19], [22]–[24]. In septic ALI, pulmonary microvascular dysfunction is the result of direct interaction of MVEC with activated PMNs, as well as the action of multiple inflammatory mediators (e.g. LPS, cytokines, and increased nitric oxide (NO) production following enhanced expression of inducible NO synthase) [10], [11], [13], [25]–[37]. Indeed, our previous work demonstrated that in septic mice, pulmonary microvascular albumin leak and oxidant stress were dependent on the presence of PMNs and mediated through CD18- and iNOS-dependent signaling [10].

Although many individual factors have been identified, the specific mechanism(s) regulating septic pulmonary microvascular, specifically MVEC, dysfunction *in vivo* remain to be determined. Sepsis-induced MVEC death, possibly through apoptosis, could lead to endothelial dysfunction, as apoptosis has been demonstrated to occur in multiple endothelial cell subtypes *in vitro* [38]–[41]. Additionally, *in vivo* manipulation of different mediators of apoptosis in animal models of sepsis, including the Fas-Fas ligand pathway, have been shown to decrease lung injury, suggesting the potential importance of apoptosis in septic organ injury [42]. The presence and specific contribution, however, of pulmonary MVEC apoptosis to sepsis-induced ALI and pulmonary microvascular dysfunction and the specific role for PMNs in this process remain to be determined.

Thus, to expand on our previous studies, we investigated the presence and role of pulmonary cell death *in vivo* in murine sepsis-induced ALI, the apoptotic nature of this cell death, MVEC involvement, as well as the specific contribution of iNOS and PMNs. In a murine cecal ligation/perforation (CLP) model of sepsis, pulmonary microvascular Evans blue (EB)-labeled albumin leak was associated with significant pulmonary cell death *in vivo* as visualized by intravital videomicroscopy (IVVM) and confirmed by histology. Septic pulmonary cell death, which was localized predominantly to MVEC and appeared to be due to apoptosis, was mediated through PMN- and CD18-dependent interaction and iNOS-dependent signaling.

## Materials and Methods

### Ethics Statement

All experimental procedures conform to the Canadian Council on Animal Care guidelines for the care and handling of animals and the institutional animal research committee at Western University approved all studies (Animal Protocol Number: #2007-002 and #2011-026).

### Animals

Male C57Bl/6 and *Nos2^−/−^* mice (8–10 weeks, 25–30 grams; Charles River, St. Constant, Quebec) were randomized to sham vs. volume-resuscitated cecal ligation and perforation (CLP)-sepsis under inhaled isoflurane anesthesia [9], [11], [43]. At 4 h, mice were prepared for pulmonary IVVM or sacrificed (pentobarbital) for collection of blood and lung tissue.

In a subset of experiments, mice were treated with cyclophosphamide by intraperitoneal injection (250 µg/g body weight) 72 h before randomization, in order to deplete circulating leukocytes. An additional cohort of mice was injected with monoclonal rat anti-mouse CD18 (ß2 integrin) immunoglobulin G2a (IgG2a) or isotype-control rat IgG2a antibody (0.4 µg/g body weight; Clone: M18/2; BD Pharmingen, Mississauga, ON) via the penile vein at the time of CLP surgery. This antibody was initially developed for use in immunohistochemistry but has been reported in a number of studies to block CD18 function [10], [44], [45].

### Pulmonary Microvascular Albumin Leak

Pulmonary microvascular leak was assessed via Evan’s blue (EB) dye technique as previously described [10]. Briefly, EB (50 µg/g) was injected into the tail vein 30 min prior to sacrifice. Pulmonary circulation was flushed with 10 mL PBS, and the lungs excised and rinsed in PBS before being snap frozen in liquid nitrogen. Frozen tissue was homogenized in ice-cold PBS, incubated with formamide at 60°C for 16 h, and centrifuged at 7,000 g for 25 min. Absorbance (A_620_ and A_740_) of the supernatant was collected and tissue EB content (µg EB/g lung/minute) was calculated.

### Pulmonary IVVM

Pulmonary IVVM was performed as previously described [9], [46]. Briefly, a 10 mm diameter transparent window was implanted in the right thoracic wall of tracheotomized, mechanically ventilated mice (Harvard rodent respiration pump, model-683, Harvard Apparatus, South Natick, MA). Pulmonary IVVM images were obtained with a long working distance 32×10.4 objective (DIAPHOT- 300 epi-fluorescence microscope, Nikon Inc., Melville NY), observed on a monitor, and recorded (Pixelfly QE High Performance Monochrome Digital Camera System with ICX-285 CCD, Opticon Corporation Ltd., Kitchener, ON). Three random digital images of the lung microcirculation per animal were captured for analysis.

### Quantification of Pulmonary PMN Sequestration and Non-viable Cells

Pulmonary microvascular PMN sequestration and non-viable cells were quantified using both IVVM and histological techniques. Blood PMNs were labeled *in vivo* following an intravenous bolus of Rhodamine-6-G (9 nmol/g body weight, Sigma) given by penile vein injection 3.5 h after sham or CLP surgery. PMN sequestration was then visualized by IVVM fluorescence microscopy (Leitz-N2 filter block; excitation/emission 530–560/580 nm). PMNs stationary in the field for >10 seconds were counted to obtain the number of sequestered PMNs per low-power field (32x).

To visualize non-viable cells in the pulmonary microcirculation, propidium iodide (PI; 0.5 µg/g body weight; Sigma) was given intravenously immediately prior to IVVM. Non-viable cells were then imaged by IVVM fluorescence microscopy (PI excitation/emission: 530/590 nm).

IVVM results were confirmed using histological techniques. Lungs were isolated and fixed in 4% PBS-buffered paraformaldehyde for 24 h. Once fixed, lungs were embedded in paraffin and sectioned (5 µm). Lung sections were then stained with Hoechst 33342 (3 sections per mouse) and 3 digital images captured of each lung section. To assess PMN sequestration, Rhodamine-6-G-positive PMNs were quantified (Rhodamine-6-G excitation/emission: 530/580 nm; Hoechst 33342 excitation/emission: 346/460 nm). Additionally, PMNs within lung sections were specifically labeled with rat anti-mouse Ly-6B.2 alloantigen primary antibody (Abd Serotec, Raleigh, NC) followed by staining with goat anti-rat IgG secondary antibody conjugated to fluorescein isothiocyanate (FITC; Santa Cruz Biotechnology, Inc., Santa Cruz, CA). Digital images were obtained and Rhodamine-6-G-positive/Ly-6B.2-positive cells were quantified (FITC excitation/emission: 490/525 nm).

To assess non-viable cells, the total number of PI-positive cells within lung tissue was quantified. Additionally, endothelial cells were specifically labeled *in vivo* by intravenous injection of Fluorescein-Griffonia simplicifolia lectin (5 µg/g; Vector Laboratories Inc., Burlington, ON). Endothelial cells in lung sections were also labeled with rat anti-mouse CD31 (platelet endothelial cell adhesion molecule/PECAM, BD Pharmingen) or rat anti-mouse CD34 (BD Pharmingen) primary antibody followed by staining with goat anti-rat IgG secondary antibody conjugated to FITC (Santa Cruz Biotechnology, Inc.). Digital images were obtained and PI-positive/lectin-positive, PI-positive/CD31-positive, or PI-positive/CD34-positive cells were quantified.

### Quantification of Apoptotic Cells in Lung Histologic Sections

To identify cells undergoing apoptosis, three different markers were used. Fragmented DNA was examined by terminal deoxynucleotidyl transferase dUTP nick end labeling (TUNEL; *In Situ* Cell Death Detection Kit Fluorescein, Roche Applied Science, Laval, QC), caspase activation was analyzed using an *in vivo* fluorescent molecule which binds activated caspases (FAM-FLIVO *In Vivo* Apoptosis Kit, Immunohistochemistry Technologies, Bloomington, MN), and loss of cell membrane polarization (as indicated by presence of cell surface phosphatidylserine) was assessed by Annexin V staining (Annexin V conjugated to Alexa Fluor 594, Invitrogen). A bolus of FLIVO was administered by tail vein injection 3 h post-injury (CLP or sham) according to the manufacturers protocol. Annexin V was administered by tail vein injection at 3.5 h post-injury (CLP or sham). Mice were euthanized at 4 h and the lungs isolated and processed as above. TUNEL staining was performed according to the manufacturers protocol and digital images of stained lungs were captured (Excitation/emission: TUNEL 494/521 nm; FLIVO 490/525 nm; Annexin V 590/617 nm).

### Blood-Pulmonary PMN Trafficking

Total white blood cell (WBC) count was measured by an automated system (STKS, Beckman-Coulter Electronics, Burlington, Ontario). Leukocyte differential was manually quantified following cytocentrifugation and Wright-Giemsa staining (Shandon Scientific, Cheshire, UK).

### Statistical Analysis

Data are mean±SEM. Between-group differences were assessed by t-test, One-Way ANOVA with Tukey post-hoc test, or Two-Way ANOVA with Bonferroni post-hoc test, where appropriate. p<0.05 was considered significant.

## Results

### Role of PMNs in CLP/Sepsis-induced ALI

**Figure 1:**
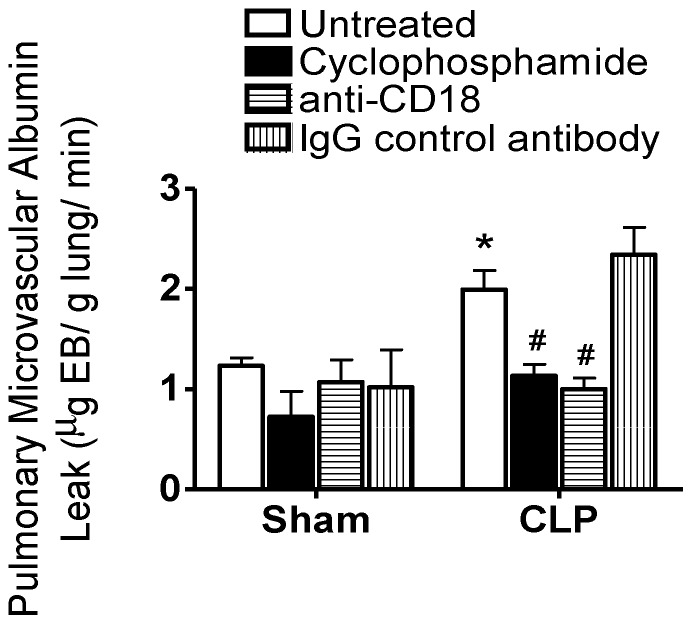
Septic pulmonary microvascular albumin leak is neutrophil (PMN)- and CD18-dependent. Cecal ligation and perforation (CLP) leads to significantly more albumin leak than sham animals; however, in mice pre-treated with cyclophosphamide or anti-CD18 antibody, albumin leak is reduced following CLP to levels observed in sham treated mice (*, p<0.05 for CLP vs. respective sham group; #, p<0.05 for Untreated CLP vs. other CLP treatment groups; Two-Way ANOVA with Bonferroni post-hoc test; n = 4 [Sham] and 6 [CLP] per treatment).

In order to confirm the induction of ALI following CLP, we assayed both pulmonary microvascular EB-labeled albumin leak and pulmonary microvascular PMN sequestration, as we previously reported [9], [11], [46]. Analysis of lung EB content revealed that CLP-treated mice had significantly more EB-albumin leak compared to sham-treated mice (Fig. 1). Cyclophosphamide-treated mice had markedly reduced circulating leukocyte levels at 72 h (Table 1), which was associated with almost complete inhibition of the CLP-induced increase in pulmonary EB-albumin leak (Fig. 1).

**Table 1:** Effect of CLP-induced Sepsis and PMN Depletion (cyclophosphamide) vs. Inhibition of PMN Adhesion (anti-CD18) on Blood Leukocyte Levels (10^9^/L).

	Untreated	Cyclo	Anti-CD18	IgG2a
Sham	1.38±0.31	0.30±0.00 #	2.38±0.78	1.05±0.13
CLP	3.38±0.74*	0.42±0.06 #	3.43±0.53	3.02±0.77

Abbreviations: PMN, neutrophil; CLP, cecal ligation and perforation; Cyclo, cyclophosphamide.

Data are mean ± SEM; n = 4 (Sham) and 6 (CLP);

*p<0.05 for CLP vs. Sham;

# p<0.05 vs. respective untreated group.

Similarly, pre-treatment of mice from the CLP group with anti-CD18 antibody, which blocks β2-integrin/CD18-dependent PMN adhesion to MVEC ICAM1, prevented the CLP/septic increase in pulmonary EB-albumin leak vs. mice from the sham group (Fig. 1). Blood leukocyte levels were slightly increased following anti-CD18 treatment (Table 1).

**Figure 2:**
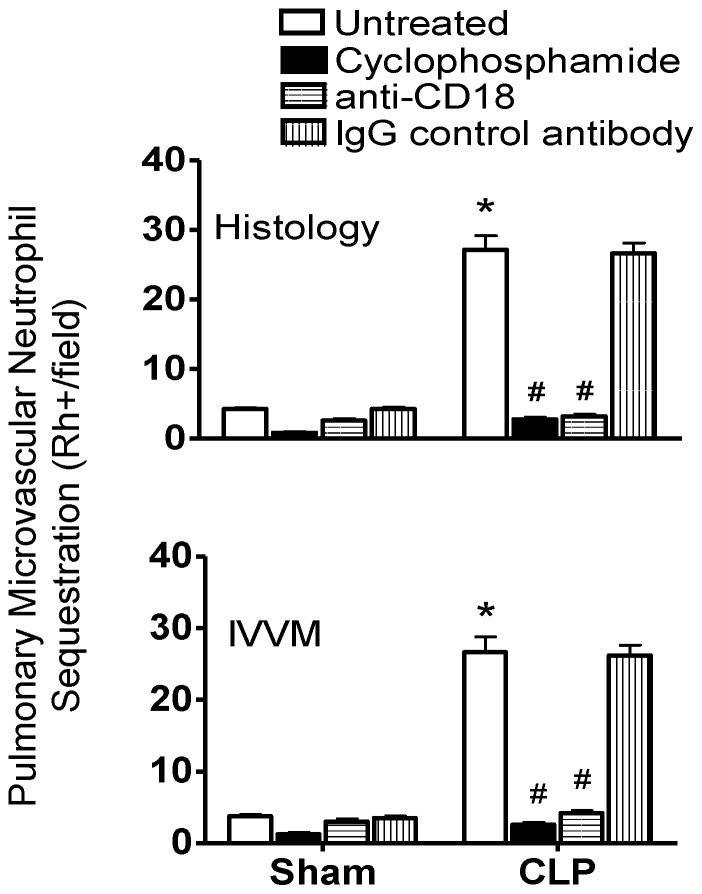
Septic pulmonary microvascular PMN sequestration is CD18-dependent. CLP enhanced pulmonary microvascular PMN sequestration, as quantified by fluorescent labeling in fixed lung sections (histology, top graph) and in lungs of live animals (intravital videomicroscopy, IVVM, bottom graph). Septic pulmonary microvascular PMN sequestration was completely inhibited by pre-treatment with cyclophosphamide or anti-CD18 antibody, but not IgG control antibody (*, p<0.05 for CLP vs. respective sham group; #, p<0.05 for Untreated CLP vs. other CLP treatment groups; Two-Way ANOVA with Bonferroni post-hoc test; n = 4 [Sham] and 6 [CLP] per treatment).

**Figure 3:**
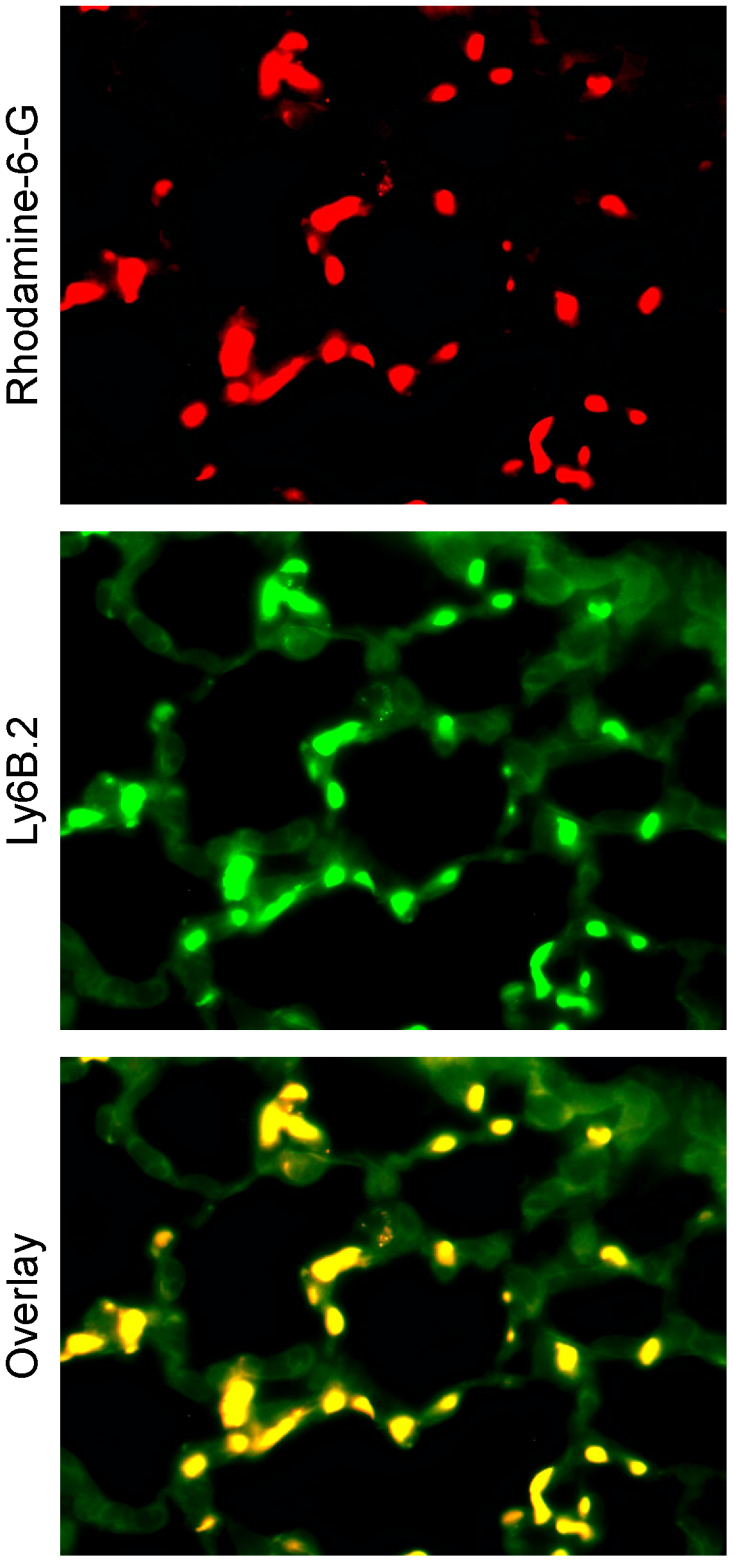
Rhodamine-6-G specifically identifies PMNs sequestered in the microvasculature of the lung under septic conditions. Cells stained *in vivo* with Rhodamine-6-G (top panel) were also detected within lung tissue sections using an antibody specific for PMNs, Ly6B.2 (middle panel) with 99.1±0.4% overlap (bottom panel).

Examination of Rhodamine-6-G-labeled PMNs sequestered in the pulmonary microvasculature *in vivo* by IVVM and *ex vivo* in lung histologic sections by immunofluorescence indicated that CLP/sepsis caused significantly more PMN sequestration compared to sham surgery (Fig. 2). Furthermore, depletion of circulating leukocytes with cyclophosphamide and blocking PMN-MVEC adhesion with anti-CD18 markedly inhibited the CLP/sepsis-induced increase in pulmonary microvascular PMN sequestration (Fig. 2). Immunohistochemical analysis of lung sections with an antibody specific for PMNs found that 99.1±0.4% of Rhodamine-6-G stained cells also stained positive for Ly-6B.2 alloantigen, which confirmed that within the pulmonary microvasculature, Rhodamine-6-G was specifically staining PMNs (Fig. 3).

### Presence of Pulmonary MVEC Death in CLP/Sepsis-induced ALI

**Figure 4:**
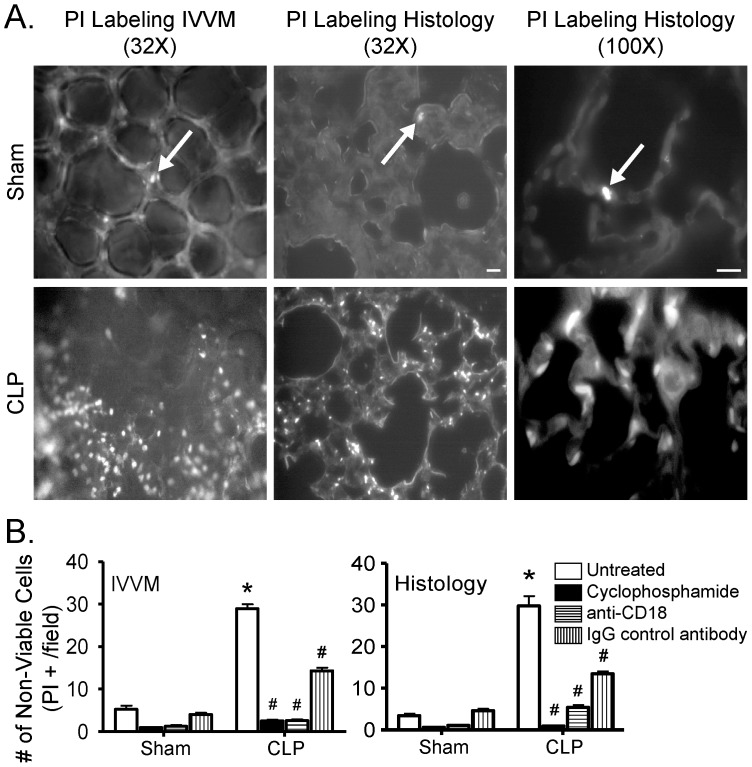
CLP/sepsis is associated with increased numbers of non-viable pulmonary cells by histology and fluorescent IVVM, and this pulmonary cell death is PMN- and CD18-dependent. (A) Nuclear PI staining identifies non-viable (dead) cells (indicated by arrows), which were much more common in mice from the CLP (bottom row) vs. sham group (top row). Scale bars: 100 µm (32X, left and middle panels) and 50 µm (100X, right panels). (B) Quantification of PI positive cells revealed a significant increase in the number of non-viable pulmonary cells following CLP, as assessed in the lungs of live mice (IVVM, left graph) and in fixed lung sections (histology, right graph). The septic increase in pulmonary non-viable cells was eliminated by pre-treatment with cyclophosphamide or anti-CD18 antibody (*, p<0.05 for CLP vs. respective sham group; #, p<0.05 for Untreated CLP vs. other CLP treatment groups; Two-Way ANOVA with Bonferroni post-hoc test; n = 4 [Sham] and 6 [CLP] per treatment).

We next assessed whether murine CLP/septic ALI was associated with evidence of pulmonary cell death *in vivo*. Fluorescent lung imaging with IVVM, following intravenous administration of the nuclear stain propidium iodide (PI), demonstrated a marked increase in PI-labeled non-viable cells *in vivo* in mice from the CLP group vs. sham group (Fig. 4A). Histological analyses confirmed this increase in PI-positive cells following CLP. In both IVVM fields and histological sections, PI-positive cells were relatively evenly distributed throughout the injured lung, and a similar number of alveoli were visualized in each field of view in mice from the CLP group vs. sham group (20.3±0.7 vs. 19.8±0.3, P = NS; 32x power).

Quantification of the number of PI-positive cells identified by either IVVM or histological techniques revealed that there was a significant increase in non-viable pulmonary cells in CLP/sepsis vs. sham mice (Fig. 4B). Cyclophosphamide treatment-induced depletion of circulating leukocytes greatly attenuated this CLP/sepsis-induced increase in the number of PI-positive cells. Furthermore, anti-CD18 inhibition of PMN-MVEC adhesion also inhibited the increase in cell death during sepsis. Together, these data suggest that pulmonary cell death is markedly increased following sepsis. Furthermore, this septic cell death is dependent on PMN presence and mediated through CD18-dependent PMN-MVEC adhesion.

**Figure 5:**
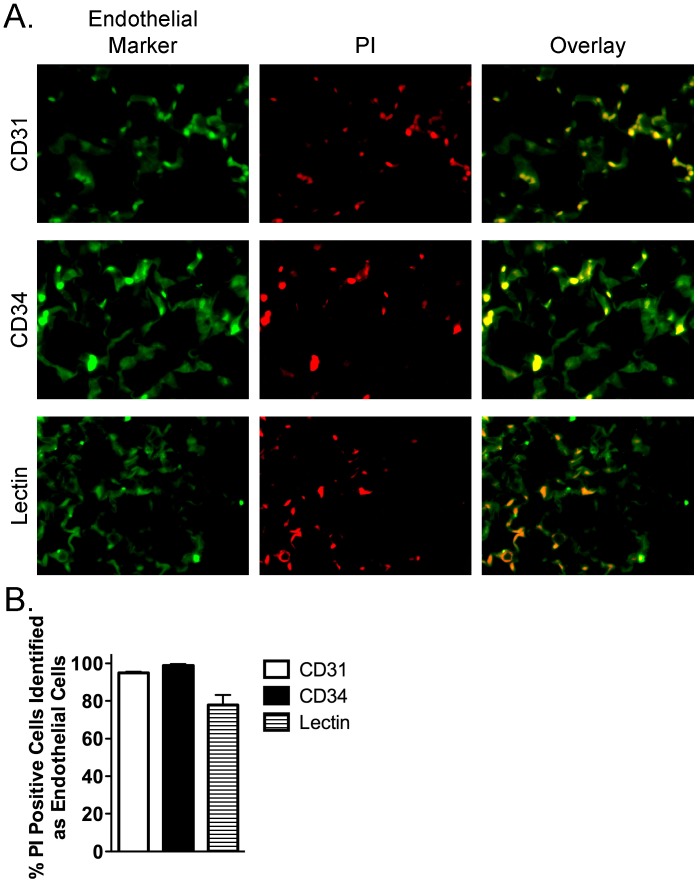
Confirmation that CLP/sepsis-induced non-viable (PI-positive) pulmonary cells are pulmonary microvascular endothelial cells. (A) Endothelial cells (left column of panels; green) were labeled with specific markers, including CD31 (PECAM; top left panel), CD34 (middle left panel), and lectin (lower left panel) in histologic pulmonary sections. Nuclear PI (red) staining identifies non-viable cells (middle column of panels). Overlap between cells positive for endothelial markers and PI (yellow) specifically identifies non-viable endothelial cells (right column of panels). (B) Quantification of the percentage of PI-positive cells that also stain positive for markers of endothelial cells confirms that the majority of CLP/sepsis-induced PI-positive/non-viable pulmonary cells are MVEC. All images 63X.

Lung sections from CLP and sham-treated mice were stained with anti-CD31 or anti-CD34 antibodies, specific endothelial cell markers. Overlay of the nuclear PI-positive signal with CD31- or CD34-immunofluorescence revealed that the septic increase in pulmonary cell death in CLP-treated versus sham-treated mice occurred almost exclusively in endothelial cells (Fig. 5A). Quantitative analysis of the overlap revealed that 95.0±0.7% of PI-positive cells were also CD31-positive and that 98.9±0.7% of PI-positive cells were also CD34-positive (Fig. 5B). To confirm these findings, endothelial cells were specifically labeled *in vivo* with fluorescein-labeled Griffonia simplicifolia lectin prior to sacrifice (Fig. 5A). Similar to our findings with CD31- and CD34-labeling, septic pulmonary PI-positive cells were mainly lectin-positive (77.9±5.3%), confirming that septic ALI was associated with pulmonary MVEC death (Fig. 5B).

**Figure 6:**
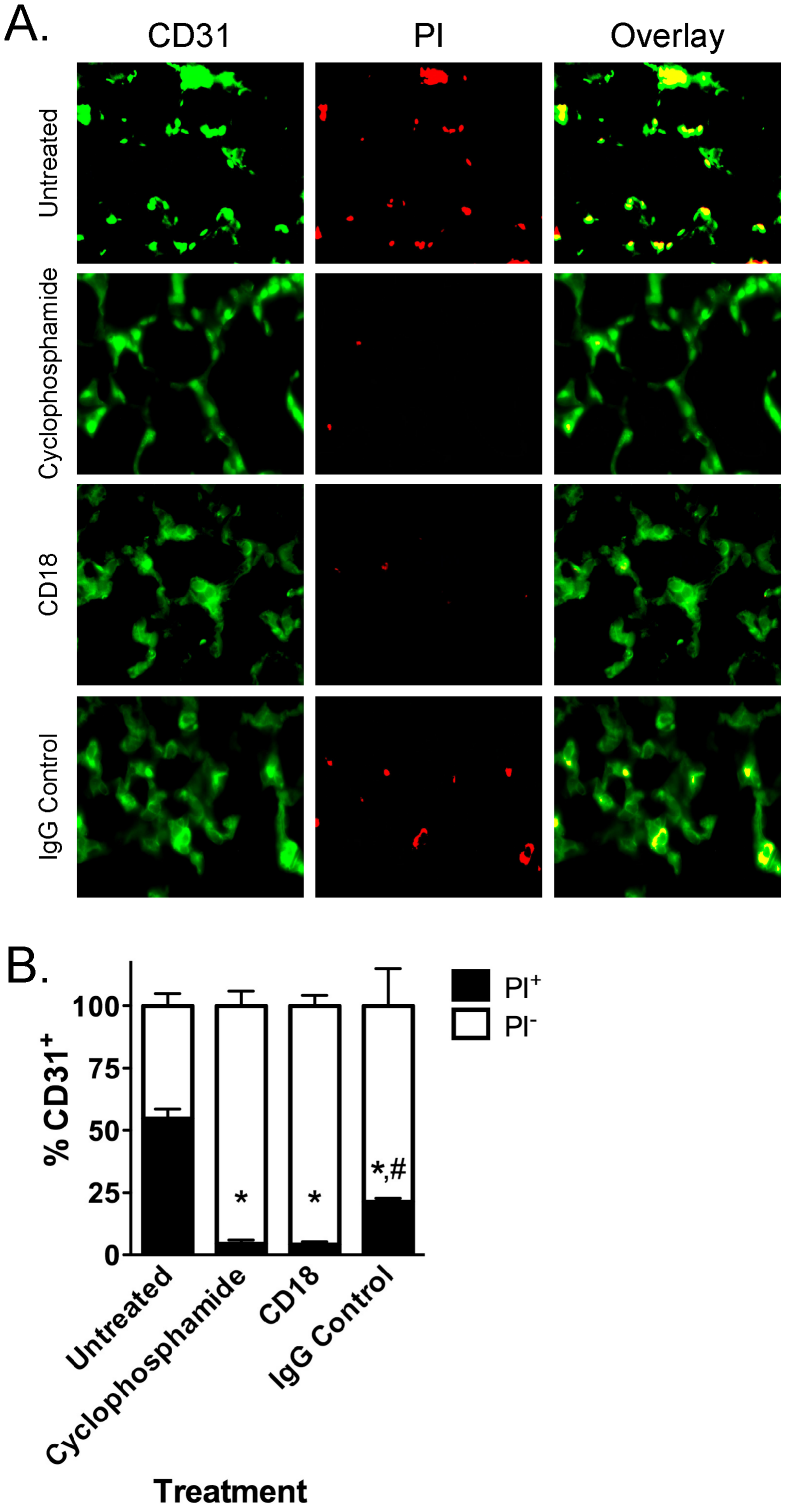
Decreased pulmonary cell death in septic mice treated with cyclophosphamide or anti-CD18 antibodies is due to a specific decrease in pulmonary MVEC death. (A) Endothelial cells were labeled with CD31 (left column of panels; green) and non-viable cells were stained with PI (red; middle column of panels). Overlap between cells positive for CD31 and PI (yellow) specifically identifies non-viable endothelial cells (right column of panels). (B) Quantification of the percentage of CD31-positive cells that are also PI-positive illustrates that treatment of septic mice with cyclophosphamide or anti-CD18 antibodies decreases the percentage of CD31-positive cells that are also PI-positive compared to untreated septic mice (*, p<0.05 for Untreated vs. other treatment groups; #, p<0.05 for anti-CD18 antibody treated vs. IgG control antibody treated; One-Way ANOVA with Tukey post-hoc test; n = 3–6 per treatment).

We used one of the EC markers, CD31 staining, to confirm that decreases in pulmonary cell death in cyclophosphamide- and anti-CD18 antibody-treated septic mice were specifically due to decreases in PI-positive pulmonary MVEC death (Fig. 6A). Quantification of the overlap between CD31-positive and PI-positive cells revealed that in septic mice, 54.7±6.3% of pulmonary MVEC (CD31-positive) were also PI-positive (Fig. 6B). In sharp contrast, in septic mice treated with cyclophosphamide or anti-CD18 antibodies, only 4.5±1.6% and 4.2±1.2%, respectively (p<0.05 for each vs. untreated septic mice) of CD31-positive cells were also PI-positive (Fig. 6B). Treatment of septic mice with IgG control antibody was also associated with a reduction in MVEC death to 21.2±1.6% (p<0.05 vs. untreated septic mice).

### Role of iNOS in CLP/Sepsis-induced Pulmonary Cell Death

**Figure 7:**
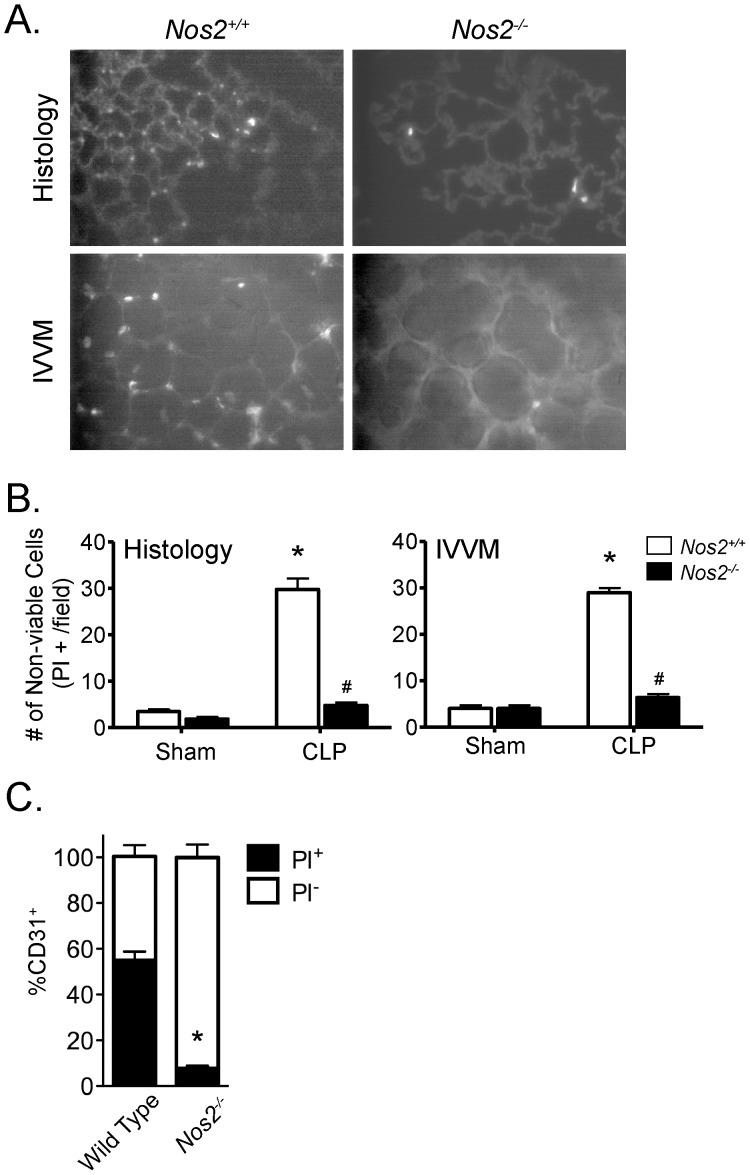
The CLP/septic increase in non-viable pulmonary cells is dependent on the presence of inducible nitric oxide synthase (iNOS). (A) The septic increase in non-viable pulmonary cells in wild type (*Nos2^+/+^*) mice (left panels), as assessed by histology (top) and by IVVM (bottom panels) was eliminated in mice lacking iNOS (*Nos2^−/−^*; right panels). (B) Quantification of the PI-positive cells revealed a significant increase in the number of non-viable pulmonary cells in *Nos2^+/+^* mice following CLP compared to *Nos2^+/+^* mice in the sham group or *Nos2^−/−^* mice in either the CLP or sham groups (*, p<0.05 for CLP vs. respective naive group; #, p<0.05 for *Nos2^+/+^* vs. *Nos2^−/−^*; two-Way ANOVA with Bonferroni post-hoc test; n = 4 [Sham] and 6 [CLP] per genotype). (C) Quantification of CD31-positive and PI-positive cells revealed that sepsis in wild type (*Nos2^+/+^*) mice was associated with marked pulmonary MVEC death (PI-positive), which was significantly attenuated in septic *Nos2^−/−^* mice (*, p<0.05 for wild type vs. *Nos2^−/−^*; t-test; n = 3–4 per genotype).

We have previously demonstrated that iNOS plays an important role in multiple features of septic lung injury in the murine CLP/sepsis model, including pulmonary microvascular endothelial barrier dysfunction and the resulting hyper-permeability [9], [10], [30], [36]. Thus, we next examined whether iNOS was required for septic pulmonary cell death in CLP vs. sham-treated mice. The CLP/sepsis-induced increase in pulmonary cell death (PI-positive cells) in wild type mice, by both IVVM and histological assessment, was completely inhibited in mice lacking iNOS (*Nos2^−/−^*; Fig. 7A and B). Furthermore, staining with CD31 confirmed that the reduction in pulmonary cell death in septic mice lacking iNOS compared to wild type mice was specifically due to a decrease in septic PI-positive pulmonary MVEC death (7.7±1.1% vs. 54.7±6.3%, respectively, p<0.05; Fig. 7C). We also confirmed our previous findings that septic increases in pulmonary microvascular EB-albumin leak and PMN sequestration (detected by both IVVM and histology) were iNOS-dependent (Table 2).

**Table 2:** Effect of CLP-induced Sepsis and iNOS/Nos2 Genotype on Parameters of Lung Injury.

	Sham		CLP	
	*Nos2^+/+^*	*Nos2^−/−^*	*Nos2^+/+^*	*Nos2^−/−^*
*Pulmonary Microvascular Protein Leak (µg EB/gram lung/min)*				
	1.2±0.1	0.8±0.1	2.0±0.2*	0.9±0.2#
*Pulmonary Microvascular PMN Sequestration (per high-power field)*				
IVVM	3.8±0.3	3.3±0.3	26.7±2.2*	7.3±0.3#
Histology	4.3±0.2	3.0±0.0	27.1±2.0*	11.0±2.0#

Abbreviations: CLP, cecal ligation and perforation; EB, Evan’s blue; PMN, neutrophil; IVVM, intravital videomicroscopy.

Data are mean ± SEM; n = 4 (Sham) and 6 (CLP);

*p<0.05 for CLP vs. Sham;

# p<0.05 for *Nos2^+/+^* vs. *Nos2^−/−.^*

### Septic Pulmonary MVEC Death is Due to Apoptosis

**Figure 8:**
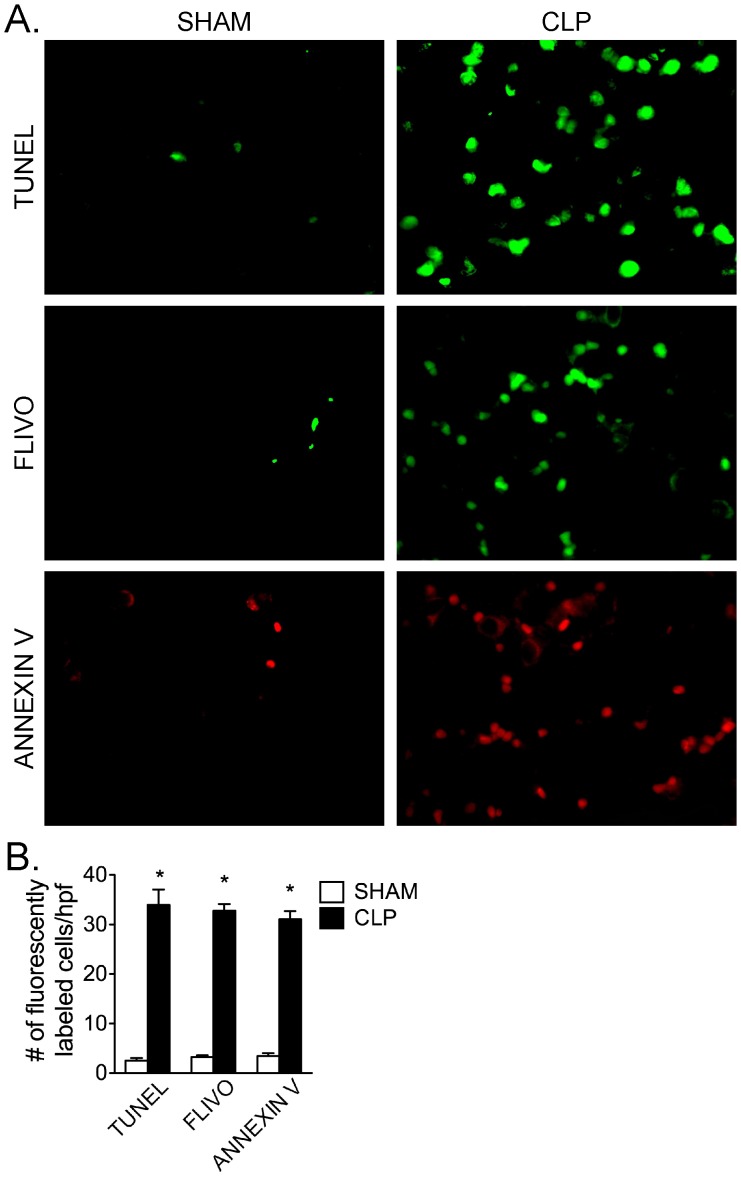
Characterization of CLP/sepsis-induced increase in non-viable pulmonary cells as apoptotic cell death by three independent markers of apoptosis. (A) DNA fragmentation was detected by TUNEL staining (top panels), caspase activation was detected by FLIVO staining (middle panels), and loss of cell membrane polarity was detected by annexin V staining (bottom panels). (B) Quantification revealed that each marker was significantly increased to a similar level in CLP vs. sham-treated mice (*, p<0.05 for CLP vs. sham group for each marker; t-test; n = 4 [Sham] and 6 [CLP] per group). All images 63X.

Finally, we assessed whether the observed septic pulmonary MVEC death was due to increased apoptosis, by examining lung histologic sections from CLP and sham-treated mice for the presence of three different markers of apoptosis: (1) DNA fragmentation (TUNEL staining); (2) caspase activation (FLIVO staining); and (3) loss of cell membrane polarity (Annexin V staining). CLP/sepsis was associated with a significant increase in the level of each of these three markers in the lungs of apoptosis vs. sham-treated mice (Fig. 8A and B). Furthermore, lung sections stained with anti-PECAM antibody demonstrated that cells positive for FLIVO were also positive for PECAM (100.0±0.5%). Lung tissue labeling with both FLIVO and Annexin V, early markers of apoptotic cells, revealed a very high degree of overlap (96.7±1.8%), which strongly suggests that septic MVEC death was most likely due to apoptosis. Analysis of CLP/septic lung tissues double-stained with FLIVO and TUNEL, however, showed a significantly lesser degree of overlap (70.3±5.3%), which may be consistent with the different apoptotic targets of FLIVO and TUNEL, or of differences in the time course of apoptotic changes detected by these markers.

## Discussion

Our study demonstrates that septic pulmonary microvascular barrier dysfunction and resulting albumin hyperpermeability in CLP/septic mice *in vivo* was associated with significant pulmonary MVEC death, as visualized by pulmonary IVVM and confirmed by histological assessment. Using cyclophosphamide-mediated PMN depletion, a commercial monoclonal antibody developed for immunodetection of murine CD18 to antagonize leukocyte adhesion *in vivo*, or *Nos2^−/−^* mice, we demonstrated reduced pulmonary MVEC death and attenuation of this septic microvascular albumin leakage. Finally, this septic pulmonary MVEC death appeared to be largely due to apoptosis, as assessed *ex vivo* by Annexin V binding, caspase activation (FLIVO labeling), and TUNEL stain. Together, our results suggest that apoptosis of the pulmonary MVEC during sepsis may be a result of leukocyte activation, and in turn, may contribute to microvascular albumin leakage during sepsis. Within this study, we have also demonstrated the use of a new approach to directly image pulmonary MVEC death *in vivo* using our IVVM technique and propidium iodide, a fluorescent marker of cell death.

Sepsis is a systemic inflammatory response due to infection, which has been shown to target multiple components of the cardiovascular system including the microvasculature and MVEC [15]. MVEC are integral to homeostatic microvascular and organ function. This is demonstrated by the ability of MVEC to modulate vascular tone, maintain a tight permeability barrier, prevent PMN adhesion and diapedesis, and reduce microvascular thrombosis [15]–[18]. Septic MVEC dysfunction leads to a loss of permeability barrier function and tissue edema [9]–[14]. Ultimately, this tissue edema results in multiple organ dysfunction/failure often leading to sepsis-induced mortality [19], [20]. Thus, understanding the mechanisms regulating septic MVEC dysfunction is critical to our understanding of sepsis and potential novel treatment options.

PMNs have been linked by multiple studies to microvascular endothelial dysfunction and the associated protein-rich pulmonary edema present during sepsis [10], [34], which we confirmed in the current study through the use of cyclophosphamide-mediated PMN depletion. Furthermore, many potential mechanisms have been proposed for PMN induction of endothelial dysfunction including PMN-MVEC physical interaction, release of PMN mediators (i.e. oxidants and proteases), and release of PMN extracellular traps [47], [48]. In the present study, using a commercial monoclonal antibody against CD18 developed for immunostaining but shown to block CD18 function [10], [44], [45], we confirmed our previous finding that septic pulmonary microvascular albumin leak is mediated through CD18-dependent interaction, and further illustrated the importance of PMN-MVEC physical interaction in initiating endothelial dysfunction.

Our data represent new studies performed to expand on our previous work by demonstrating *in vivo* that PMN-dependent septic pulmonary microvascular barrier dysfunction is associated with significant pulmonary MVEC death, which is largely apoptotic. Death of endothelial cells, possibly due to apoptosis, has long been proposed as a mechanism of septic endothelial dysfunction; however, direct *in vivo* evidence in a clinically relevant model of sepsis (e.g. CLP) has been difficult to obtain. In fact, only a few studies have reported the presence of apoptotic endothelial cells under septic conditions [49]–[52], which may be due to shedding of apoptotic endothelial cells from the vasculature [40]. Most of these studies, however, primarily used intravenous instillation of endotoxin as a “septic” model, used only a single marker of apoptosis (TUNEL staining), or did not use specific markers to confirm that apoptotic cells were indeed endothelial cells.

The optimal marker for apoptosis remains controversial and in many cases, appears to be cell type specific. Thus, we assessed apoptosis using three distinct markers of apoptosis: surface phosphatidylserine localization (Annexin V), caspase activation (FLIVO), and DNA fragmentation (TUNEL). Furthermore, to confirm the identity of the PI-positive/dead pulmonary cells, which were largely apoptotic cells, we used three discrete endothelial cell specific markers: CD31 (PECAM), CD34, and Griffonia simplicifolia lectin. Importantly, our study is consistent with previous studies, but our data also further develop the concepts from these studies by demonstrating an association between septic pulmonary microvascular albumin leak due to MVEC dysfunction and pulmonary MVEC death, putatively largely due to apoptosis. Moreover, we illustrate the importance of PMNs in the induction of this septic pulmonary MVEC death through apoptosis.

Multiple studies have further examined endothelial cell apoptosis *in vitro* to identify important regulatory pathways in response to sepsis. These studies have identified many mediators of endothelial cell apoptosis including caspase activation, calpain 1 activity, and signaling by cytokines from the TNF superfamily [41], [51], [53]–[56]. Furthermore, of these pathways, both caspase activation and TNF superfamily associated pathways have also been shown to mediate the endothelial response *in vivo* [42], [51], [54]. The increased pulmonary microvascular FLIVO staining (marker of caspase activation) we observed in mice from the CLP group is consistent with these findings, and this integral role for caspase activity in mediating sepsis-induced pulmonary MVEC death would be consistent with predominantly apoptotic MVEC death.

We also demonstrate that both CD18-dependent PMN-MVEC adhesion and iNOS are essential to CLP/sepsis-associated pulmonary cell death. PMN induction of endothelial cell death has been demonstrated previously in both *in vitro* and *in vivo* models [57]–[59]. In fact, in a model of diabetic retinopathy, Joussen and co-workers demonstrate that CD18-dependent PMN-endothelial cell interaction is required for endothelial cell injury and PI-positive death [57], findings that reinforce our putative mechanism of septic PMN-dependent pulmonary MVEC death through apoptosis. These authors, however, only identified non-viable endothelial cells, and did not specifically examine markers of apoptosis.

Interestingly, while injection of anti-CD18 antibodies abolished septic MVEC death, we found that injection of isotype (IgG) control antibodies also significantly decreased MVEC death. It is noteworthy that murine FC receptors have been previously shown to non-specifically bind to IgG from multiple species [60], [61]. As such, we speculate that in the present study, binding of isotype control antibody to FC receptors on PMNs may have resulted in enhanced clearance of the PMNs, resulting in the observed reduction in septic pulmonary MVEC death.

Increased NO production due to enhanced iNOS expression/activity in organs/vasculature and in individual cells (i.e. PMNs) has been demonstrated in both animals and humans with sepsis [25], [29]. PMNs are an important source of iNOS-derived NO in sepsis as many PMN actions are NO-dependent [10], [62]. Our previous studies have revealed a specific role for PMN iNOS in septic pulmonary microvascular endothelial dysfunction [34]. Here we show that iNOS also mediates septic pulmonary cell death, putatively MVEC apoptosis, as mice lacking iNOS have significantly less pulmonary cell death compared to wild type mice following CLP. It remains to be determined, however, whether this iNOS-dependent septic pulmonary cell death, which is largely apoptotic, is specifically mediated by PMN iNOS, which is the subject of ongoing studies.

iNOS has been found to promote apoptosis in multiple organs/cell types. Mice lacking iNOS or treated with a synthetic inhibitor of iNOS were reported to have decreased numbers of apoptotic alveolar and bronchiolar epithelial cells following sepsis, a finding similar to our study [63]. Additionally, iNOS was shown to enhance/induce apoptosis *in vitro* in cardiac myocytes and in vascular endothelial cells in response to cytokines, and *in vivo* in models of spinal cord injury and intestinal ischemia-reperfusion [56], [64]–[66]. Together, these studies along with our results highlight the potential importance of iNOS in promoting CLP/sepsis-induced pulmonary cell death, which is putatively MVEC apoptosis.

In conclusion, septic ALI is associated with pulmonary microvascular barrier dysfunction and pulmonary MVEC death. This pulmonary MVEC death was predominantly due to apoptosis, was dependent on the presence of PMNs, and was mediated through CD18- and iNOS-dependent signaling. Our results suggest that septic apoptosis of the pulmonary MVEC may be a result of leukocyte activation and iNOS-dependent signaling, and in turn, may contribute to pulmonary microvascular barrier dysfunction and albumin hyper-permeability in sepsis-induced lung injury. Future studies will focus on identifying the specific cellular source of iNOS (i.e. PMN vs. MVEC), understanding the molecular mechanisms responsible for the induction of septic pulmonary MVEC apoptosis, and exploring the potential therapeutic importance of this pathway.
